# The Multifaceted Role of Oxytocinergic System and *OXTR* Gene

**DOI:** 10.1055/s-0044-1779039

**Published:** 2024-01-18

**Authors:** Rakibul Hasan

**Affiliations:** 1 Department of Biomedical Engineering, Case Western Reserve University, Cleveland, Ohio, United States

**Keywords:** oxytocin, *OXTR* gene, social behavior

## Abstract

The article explores the multifaceted role of the neuropeptide oxytocin in human behavior and its connection to the oxytocin receptor ( *OXTR* ) gene. Oxytocin, produced in specific brain nuclei, is implicated in emotional, social, and maternal behaviors, stress reduction, uterine contraction during childbirth, and lactation. The *OXTR* gene, located on chromosome 3, encodes oxytocin receptors found in various body parts, including critical brain regions associated with social behaviors. The article delves into studies on rodents, revealing correlations between *OXTR* gene expression and pair bonding in the prefrontal cortex and social behavior regulation in the amygdala. The discussion extends to the impact of oxytocin on social support-seeking behavior, focusing on a specific genetic variation, rs53576. The article explores how this genetic variation influences empathy, stress reactivity, and susceptibility to disorders such as autism and social anxiety. Furthermore, the article examines structural and functional changes in the brain associated with *OXTR* gene variations. It discusses the role of DNA methylation in influencing oxytocin receptor availability, affecting social perception and responsiveness to negative stimuli. The article also highlights the oxytocinergic system's involvement in disorders such as autism and social anxiety, emphasizing the interplay between genetics and environmental factors. The article also touches on the potential therapeutic use of exogenous oxytocin in mitigating symptoms associated with these disorders. In summary, the article underscores the intricate relationship between oxytocin, the *OXTR* gene, and diverse aspects of human behavior, providing insights into social bonding, perception, and the development of behavioral disorders.

## Introduction

Neuropeptide oxytocin, produced in hypothalamic supraoptic and paraventricular nuclei, is associated with emotional and social behaviors and maternal bonding. 1 Oxytocin facilitates social behavior, fosters pair bonding, increases social interaction, generosity, eye gaze, empathy, and trust and attachment in humans and animals. 2 Oxytocin is also involved in the reduction of stress, 3 contraction of the uterus during childbirth, 4 and lactation after childbirth. 5 The effect of oxytocin on human behavior depends on its binding to oxytocin receptors located in different parts of the human body. 6 Oxytocin receptor is a cell surface membrane receptor that belongs to class I G protein-coupled receptor family. 7 Oxytocin receptor ( *OXTR* ) gene is located in chromosome 3 of the human genome. 7 *OXTR* gene spans 19.2 kilobases on chromosome 3, encodes 389 amino acids, contains three introns and four exons, and expresses in reproductive structures, prefrontal cortex, amygdala, hypothalamus, and olfactory nucleus. 8 9 Oxytocin receptors have also been found in the hippocampus, periaqueductal gray, striatum, nucleus accumbens, ventral tegmental area, lateral septum, medial preoptic area, etc. 10 Findings from animal studies suggest that *OXTR* gene expression in the prefrontal cortex is associated with pair bonding, maternal and anxiety-related behavior. 11 12 13 On the other hand, *OXTR* gene expression in the amygdala is associated with the regulation of social behavior and fear expression. 14 15 In this article, I plan to discuss the importance of *OXTR* gene and its variations in determining social behavior, social perception, associated changes in brain structure and functionalities, and tendency to develop brain disorders associated with variants of this gene.

## Effects on Maternal and Pair-Bonding Behavior

Oxytocin is involved in uterine contraction during childbirth. Around the onset of the labor, *OXTR* gene transcription increases which results in an increase in the number of OXTR messenger RNAs (mRNAs) and oxytocin receptors in the myometrium of the uterus. 4 As a result, more oxytocin can bind to its receptor in the uterus which facilitates uterine contraction. Along with myometrium, *OXTR* gene expression also increases in human choriodecidual tissue around the onset of the labor. 16 After parturition, the number of oxytocin receptors rapidly declines. In rats, the number of oxytocin receptor mRNAs decreases sevenfold within 24 hours after parturition which results in the downregulation of oxytocin receptors in the uterus. 17 After childbirth, oxytocin regulates lactation. Oxytocin receptors are upregulated in the myoepithelial cells surrounding the milk ducts in the mammary gland during lactation, and binding of oxytocin-to-oxytocin receptors plays a key role in the ejection of milk from the mammary gland. 5 The expression of the *OXTR* gene also plays a vital role in determining the extent of maternal behavior present in a mother. In a previous study on rats, it was found that female rats who showed a greater amount of maternal behavior had a higher number of oxytocin receptors present in the medial preoptic area, lateral septum, central nucleus of the amygdala, and paraventricular nucleus of the hypothalamus. 18 Another study found that the reduction of oxytocin receptors in the medial prefrontal cortex decreased quality of maternal care. 12 Research findings suggest that such a decrease in *OXTR* gene expression happens due to epigenetic modification through DNA methylation. 19 In addition to maternal behavior, the expression of the *OXTR* gene is very important for pair bonding. Previous studies on prairie voles found that the nucleus accumbens of monogamous prairie voles has a higher density of oxytocin receptors than the nucleus accumbens of nonmonogamous prairie voles, 20 and an increase in the density of oxytocin receptors in the nucleus accumbens facilitates pair-bonding-related behavior. 21 Moreover, another study found that the infusion of oxytocin antagonists in the nucleus accumbens or prefrontal cortex reduced partner preference formation or pair bonding in female prairie voles. 20

## Effects on Social Behavior

In addition to maternal behavior and pair bonding, the oxytocinergic system plays a vital role in shaping social behavior. In our daily life, we face numerous stressful situations. To cope with stressful situations, it is necessary to seek social support. The tendency to seek social support varies from individual to individual, and variations in the oxytocin receptor gene are associated with individual differences in the tendency to seek social support. In particular, a particular single-nucleotide polymorphism (SNP) rs53576 within intron 3 of the *OXTR* gene has been associated with the tendency to seek social support in adult humans. 22 An SNP refers to a variation of nucleobases (adenine [A], guanine [G], cytosine, or thymine) in a nucleotide within a DNA sequence. For example, someone may have an A allele at a particular location in a gene, and someone else may have a G allele at that location. Everyone carries two copies of each gene (one from each parent). If that person receives either A or G allele from both parents, that individual may be referred to as homozygous A or G, respectively, for that SNP. If that person receives an A allele from one parent and G allele from the other parent, that person may be referred to as heterozygous AG for that SNP. Such variations in a single nucleotide of the *OXTR* gene may determine the number and/or distribution of oxytocin receptors in the brain. 23 The A allele of rs53576 has been linked to reduced trust, 24 greater self-reported stress, 25 and reduced responsiveness to social support in anticipation of stress. 22 Additionally, stress hormone cortisol secretion does not change significantly when people with homozygous A allele seek emotional support. 22 On the other hand, individuals with homozygous G or heterozygous AG show a higher tendency to seek emotional support 26 and secrete less cortisol in response to social support. 22 Only in these two groups, social support decreases the feelings of anxiety but not for homozygous A group. 22 These findings suggest that G allele carriers trust people more and seek help from others more often than A allele carriers. Additional studies also suggested that individuals who contain one or two copies of A allele (AA/AG) in SNP rs53576 of the *OXTR* gene are more likely to be diagnosed with autism, 27 display less empathy, 25 and show lower levels of sensitive responsiveness toward their children. 28 Overall, variations of the rs53576 genotype are very crucial in determining an individual's ability to show empathy and stress reactivity.

## Effects on Brain Structure and Functionalities

Variations of rs53576 genotype not only impact the behavior of individuals but also change the structure and function of brain regions. Tost et al (2010) found that individuals with homozygous A had lower gray matter in the hypothalamus than individuals with AG or GG phenotypes. 29 Additionally, during perceptual processing of facial emotion, individuals with AA genotype had much lower activation in the amygdala but higher functional coupling between amygdala and hypothalamus than individuals with AG/GG genotypes. 29 Previous studies also found both hypo- and hyperactivities of the amygdala during face processing are associated with autism 30 31 which is linked to the A allele of rs53576 SNP. 27 On the other hand, an increase in functional connectivity between the hypothalamus and the amygdala could be a result of deficits in the top-down modulation of the hypothalamus. 32 Overall, lack of empathy, abnormal stress reactivity, lower levels of sensitive responsiveness toward children—all these traits present in AA genotype could be linked to abnormal structure of hypothalamus, abnormal function of amygdala, and abnormal functional coupling between amygdala and hypothalamus.

## Effects on Social Perception

Oxytocin not only modulates social behavior but also influences social perception. It is involved in the processing of emotionally charged stimuli such as facial expressions. Oxytocin reduces the activity of the amygdala when viewing negatively valenced stimuli. 33 For example, in one study, after intranasal oxytocin administration, the amygdala of human subjects showed reduced activity when viewing angry faces but showed increased activity when viewing happy faces. 34 The effect of oxytocin on the human brain depends on the expression of the *OXTR* gene. Epigenetic changes such as DNA methylation of the *OXTR* gene can influence the expression of this gene. DNA methylation usually decreases gene expression. 35 Puglia et al (2015) found that higher methylation of the *OXTR* gene increased the activity of the amygdala and other brain regions associated with face perception when viewing negative facial expressions (angry and fearful faces). 36 As methylation decreases *OXTR* gene expression, the number of available oxytocin receptors for binding decreases due to methylation. As a result, the effect of oxytocin is reduced in the brain, and individuals become more responsive toward negative aspects of visual stimuli. Additionally, Puglia et al (2015) found that connectivity between amygdala and insula is higher when OXTR methylation is lower. 36 Denny et al (2014) found that higher connectivity between amygdala and insula might be associated with habituation and desensitization of repeated negative stimuli. 37 Habituation and desensitization of negative stimuli are very important to prevent the development of disordered social perception. So, increasing gene expression of the *OXTR* gene is important not only for reducing response to negative stimuli but also for preventing the development of disordered social perception.

## Effects on Developing Brain Disorders

The activity of the oxytocinergic system influences not only social behavior and social perception but also the development of brain disorders associated with social behavior, such as autism and social anxiety disorder (SAD). People with autism have severe deficits in social interaction and communication. I already mentioned that A allele in rs53576 SNP of the *OXTR* gene is linked to the development of autism. 27 Functional changes in brain areas (involved in social cognition and interaction) such as the amygdala, medial prefrontal cortex, and insula due to variations in oxytocin receptor genes could be linked to the development of autism. 38 But symptoms of autism can be alleviated through intranasal administration of oxytocin. 39 40 41 In addition to autism, the oxytocinergic system is involved in the development of SAD. In SAD, individuals have excessive anxiety or fear about negative evaluations by others in social situations. Genetic polymorphism of the *OXTR* gene plays an important role in the development of SAD. 42 For example, individuals with GG allele in SNP rs2254298 of the *OXTR* gene have higher SAD incidence rates than individuals with AG allele. 42 Similarly, as variations of rs53576 SNP determine empathetic, prosocial, and support-seeking behaviors, this SNP can also be implicated in the development of SAD. One study found that individuals with A allele in rs53576 had more concerns regarding the negative perception of one's company, 43 and another study found that insecurely attached study participants with at least one A allele showed significantly higher social anxiety than homozygous G participants. 44 So, individuals with AA/AG alleles in rs53576 are more likely to develop SAD. But some other studies have found that interaction between childhood family environment and genotype is more important than particular genetic variation in the development of SAD. 45 46 47 I mentioned before that the homozygous G allele of rs53576 promotes prosocial, support-seeking, and empathetic behaviors, and homozygous A allele does the opposite. 22 24 25 26 But one study found that homozygous G allele carriers show less resilient coping styles if they are raised in an adverse environment than homozygous A allele carriers. 45 As a result, when individuals with homozygous G allele are raised in adverse environments, they show increased social withdrawal and decreased perceived social support than homozygous A individuals. 46 Another study showed that individuals with homozygous G allele had less gray matter in the ventral striatum, a brain region involved in reward, motivation, and decision processing, when they had more traumatic experiences in childhood. 47 Reduced ventral striatum is associated with reduced responsiveness toward social support. 47 Although homozygous A allele is usually associated with reduced tendency to seek social support, 22 homozygous G individuals are more prone to develop reduced responsiveness toward social support when they are maltreated as children than homozygous A individuals. So, in a stable raising environment in childhood, homozygous G individuals are less vulnerable to develop SAD, but in an adverse raising environment in childhood, homozygous G individuals are more vulnerable to develop psychiatric disorders such as SAD than homozygous A individuals. These findings show that interaction between genes and environment is very important in the development of behavioral disorders such as SAD. To alleviate symptoms of SAD, studies have been performed to understand the effectiveness of exogenous oxytocin administration as a treatment method. Those studies found that intranasal administration of oxytocin does not alleviate symptom severity of SAD 48 but reduces the level of attentional bias toward emotional faces 49 and decreases responsiveness toward fearful faces by reducing activity of amygdala 50 and medial prefrontal cortex ( Table 1 ). 51

**Table 1:** Effects of genotype variation of SNP rs53576 within *OXTR* gene on social behavior, brain structure/functionalities, and development of brain disorders

rs53576 genotypes	AA	AG/GG
Social behavior	Reduced trust Greater self-reported stress Unchanged cortisol level when seeking emotional support Reduced responsiveness to social support Lack of empathy Lower level of sensitive responsiveness toward own children Increased concern about negative perception of own's company	Higher trust Higher tendency to seek emotional support Reduced secretion of cortisol when seeking emotional support Seeking social support decreases anxiety
Brain structure and functionalities	Lower amount of gray matter in hypothalamus During processing facial emotion, lower activation in amygdala and higher functional coupling between amygdala and hypothalamus	Higher amount of gray matter in hypothalamus During processing facial emotion, higher activation in amygdala and lower functional coupling between amygdala and hypothalamus
Brain disorders	Linked to development of autism Linked to development of social anxiety disorder	Effects of positive social behavior and perception reverted if raised in adverse environment

Abbreviations: A, adenine; G, guanine; SNP, single-nucleotide polymorphism.

## Conclusion

To sum up, the effects of oxytocinergic signals in the body and brain largely depend on the expression of the *OXTR* gene. Expression patterns of the *OXTR* gene in specific parts of the body and brain regions can control certain physiological and social behaviors in both animals and humans. Both SNP and epigenetic modification through DNA methylation can play a crucial role not only in shaping social behavior and social perception but also in developing brain disorders associated with social behavior. But in certain cases, development of behavioral disorders can depend on the interaction between the *OXTR* gene and environment. Abnormality in social behavior can be treated to some extent through administration of exogenous oxytocin which can help improve social perception and interaction.
